# Supplementary material for: The Prevalence, Genotype Distribution and Risk Factors of Human Papillomavirus in Tunisia: A National-Based Study
Source: Viruses. 2022 Sep 30;14(10):2175. doi: 10.3390/v14102175 (PMC9611589; doi:10.3390/v14102175)
Supplement: Supplementary file 1 [file viruses-14-02175-s001.zip › Table S1.pdf]

Table S1. Representation of grand-regions, regions and governorates implicated in the study

Grand-regions	Regions	Governorates	N
	Grand Tunis	TUNIS	153
Grand Tunis		ARIANA	80
		BEN AROUS	94
		MANOUBA	65
North	North-East	NABEUL	99
		ZAGHOUAN	25
		BIZERTE	82
	North-West	BEJA	39
		JENDOUBA	60
		KEF	38
		SILIANA	35
Center	Center-East	SOUSSE	80
		MONASTIR	73
		MAHDIA	53
		SFAX	121
	Center-West	KAIROUAN	80
		KASSERINE	53
		SIDI_BOUZID	59
South	South-East	GABES	53
		MEDENINE	66
		TATAOUINE	20
	South-West	GAFSA	50
		TOZEUR	18
		KEBELI	21
		Total	1517
