# Supplementary material for: The Prevalence, Genotype Distribution and Risk Factors of Human Papillomavirus in Tunisia: A National-Based Study
Source: Viruses. 2022 Sep 30;14(10):2175. doi: 10.3390/v14102175 (PMC9611589; doi:10.3390/v14102175)
Supplement: Supplementary file 1 [file viruses-14-02175-s001.zip › Table S2.pdf]

Table S2. Comparison between women with β-globin positive and β-globin negative PCR (N_T=1482)

	Positive β-globin n(%*)	Negative β-globin n(%*)	P
**Collection center(N=1482)**			
CSR	642(43.3%)	117(7.9%)	0.082
CSRS	587(39.6%)	136(9.2%)	
**Age**			
<=20	9(0.6%)	0	0.603
[20-30[	162(11%)	39(2.7%)	
[30-40[	426(29%)	95(6.5%)	
[40-50[	432(29.4%)	83(6.5%)	
[50-60[	165(11.2%)	31(2.1%)	
>60	24(1.6%)	5(0.3%)	
**Education level (N=1474*)**			
Illiterate	253(17.2%)	49(3.3%)	0.653
Primary school	490(33.2)	100(6.8)	
Basic school	91(6.2%)	21(1.4%)	
High school	272(18.5%)	55(3.7%)	
Professional	13(0.9%)	6(0.4%)	
College	104(7.1%)	20(1.4%)	
Missing data			
**Marital status(N=1482)**			
Married	68	14	1
Single (widow, divorced, never married)	1161	239	
Missing data			
**Monthly income (N=1428*)**			
< 350TDN	420(29.4%)	78(5.5%)	0.440
[350TDN-700TDN]	463(32.4%)	106(7.4%)	

>700TDN	298(20.9%)	63(4.4%)	
Missing data			
**Housing type (N=1482)**			
Traditional house	530(35.8%)	100(6.7%)	
Villa	570(38.5%)	126(8.5%)	
Apartment	110(7.4%)	21(1.4%)	0.550
Rudimentary	19(1.3%)	6(0.4%)	
Missing data			
**Occupation (N=1482)**			
Yes	360(24.3%)	63(4.3%)	0.159
No	869(58.6%)	190(12.8%)	
Missing data			
**Smoking(N=1482)**			
Yes	72(4.9%)	10(0.7%)	0.227
No	1157(78.1%)	243(16.4%)	
Missing data			
**Menopause(N=1471*)**			
Yes	235(16%)	45(3.1%)	0.624
Non	985(67%)	206(14%)	
**Pregnancy(N=1344*)**			
Yes	82(6.1%)	25(1.9%)	0.086
No	1029(76.6%)	208(15.5%)	
Missing data			
**Contraception(N=1421*)**			
Yes	699(49.2%)	143(10.1%)	0.2632
Non	475(33.4%)	104(7.3%)	
Missing data			
**Medical history (N=1477*)**			
Yes	367(24.9%)	77(5.2%)	0.875
No	854(58%)	175(11.9%)	

Missing data			
**Surgical history (N=1476*)**			
Yes	446(30.2%)	113(7.7%)	
No	778(52.7%)	139(9.4%)	
Missing data			
**STI history (N=1465*)**			
Yes	240(16.4%)	53(3.6%)	0.554
No	977(66.7%	195(13.3%)	
Missing data			
**Multiple sexual intercourse of partner (N=1450*)**			
Yes	79(5.4%)	17(1.2%)	0.778
No	110(75.9%)	227(15.7%)	
Not sure	21(1.4%)	6(0.4%)	
Missing data			
**Age of first sexual intercourse (N=1382*)**			
≤ 20 years	394(28.5%)	74(5.4%)	0.457
>20 years	755(54.6%)	159(11.5%)	
Missing data			
**Multiple sexual intercourse (N=1334*)**			
One partner	943(70.7%)	198(14.8%)	0.291
Two partners	30(2.2%)	8(0.6%)	
More than two partner	21(1.6%)	3(0.2%)	
Does not wish to reply	116(8.7%)	15(1.1%)	
Missing data			

* difference between N_T and N is due to missing data; STI: sexual transmitted infection
