# Supplementary material for: The Prevalence, Genotype Distribution and Risk Factors of Human Papillomavirus in Tunisia: A National-Based Study
Source: Viruses. 2022 Sep 30;14(10):2175. doi: 10.3390/v14102175 (PMC9611589; doi:10.3390/v14102175)
Supplement: Supplementary file 1 [file viruses-14-02175-s001.zip › Table S3.pdf]

Table S3. HPV types distribution according to cytological profile

	Normal Cytology	Cytology with benign transformation	Cytology with intraepithelial lesions
**HPV11**	1(2%)	1(2.8%)	1(10%)
**HPV59**	7(14%)	1(12.5%	0
**HPV6**	9(18%)	5(13.9)	6
**HPV40**	9(18%)	3(8.3%)	1(10%)
**HPV70**	2(4%)	2(5.6%)	0
**HPV74**	5(10%)	0	0
**HPV16**	7(14%)	4(11.1%)	0
**HPV31**	5(10%)	6(16.7%)	1(10%)
**HPV35**	1(2%)	1(2.8%	0
**HPV39**	1(2%)	1(2.8%)	0
**HPV52**	4(8%)	2(5.6%)	1(10%)
**HPV54**	1(2%)	0	1(10%)
**HPV53**	2(4%)	1(2.8%)	1(10%)
**HPV58**	0	2(5.6%)	0
**HPV62**	1(2%)	1(2.8%)	0
**HPV66**	2(4%)	1(2.8%)	0
**HPV68**	2(4%)	3(8.3%)	1(10%)
**HPV56**	0	2(5.6%)	0
**HPV42**	6	2(5.6%)	0
**HPV43**	0	0	1(10%)
**HPV44**	0	1(2.8%)	0
**HPV81**	1(2%)	1(2.8%)	1(10%)
**HPV84**	0	1(2.8%)	0
**HPV89**	1(2%)	1(2.8%)	1(10%)
**HPV18**	2(4%)	2(5.6%)	0
