# Supplementary material for: The Prevalence, Genotype Distribution and Risk Factors of Human Papillomavirus in Tunisia: A National-Based Study
Source: Viruses. 2022 Sep 30;14(10):2175. doi: 10.3390/v14102175 (PMC9611589; doi:10.3390/v14102175)
Supplement: Supplementary file 1 [file viruses-14-02175-s001.zip › Table S4.pdf]

Table S4. Univariate analysis for the identification of potential risk factors related to HPV infection in Tunisia

Variable	N	n	Prevalence %	OR	CI95%	p
**Grand-Region**						
Grand Tunis	327	43	13.1	6.4	2.8-14.6	<0.001
South	172	20	11.6	5.2	2.1-12.7	
Central	427	27	6.3	2.8	1.2-6.6	
North	303	6	2.0	Ref		
**Age group**						
<30	202	25	12.4	2.0	1.1-3.6	0.055
30-40	441	31	7.0	1.071	0.6-1.8	
>50	423	13	8.1	1.406	0.7-2.7	
40-50	160	27	6.4	Ref		
**Marital status**						
Single	68	16	23.5	4.158	2.2-7.6	<0.001
Married	1161	80	6.9	Ref		
**Professional activity**						
yes	360	40	6.4	0.551	0.3-0.8	0.006
non	869	56	11.1	Ref		
**Educational level**						
Illiterate	253	14	5.5	Ref		0.2
Primary level	490	39	8	1.476	0.8-2.7	
Secondary and high level	480	43	9	1.680	0.9-3.1	
**Housing type**						
Traditional house	530	41	7.7	0.9	0.5-1.4	0.579
Villa	570	41	7.2	Ref		
Apartment	110	12	10.9	0.6	0.3-1.2	
Rudimentary	19	12	10.5	0.6	0.1-2.9	
**Monthly household income (TND)**						
>700	289	28	9.7	1.5	0.9-2.7	0.22
350-700	472	31	6.6	1.0	0.6-1.7	
<350	420	27	6.4	Ref		
**Smoking**						
yes	1157	19	26.4	5.0	2.8-8.9	<0.0001

no	72	77	6.7	Ref		
**Medical history**						
yes	367	29	7.9	1.0	0.6-1.6	0.861
no	854	65	7.6	Ref		
**Surgical history**						
yes	446	40	9	1.2	0.8-1.9	0.232
non	778	55	7.1	Ref		
**Menopause**						
yes	235	16	6.8	0.8	0.4-1.4	0.533
no	985	79	8	Ref		
**Pregnancy**						
yes	82	8	9.8	1.3	0.6-2.7	0.5
no	1029	79	7.7	Ref		
**Contraception**						
yes	699	56	8	1.1	0.7-1.7	0.59
no	475	34	7.2	Ref		
**Gestality**						
o	45	5	11.1	1.5	0.5-3.8	0.401
>1	1171	9	7.7	Ref		
**Parity**						
0	69	10	14.5	0.3	0.1-0.7	
[1-2]	436	41	9.4	0.6	0.3-0.9	
>3	701	42	6	Ref		
**Age at first intercourse**						
<20	394	33	8.4	1.3	0.8-2.1	0.205
>=20	755	48	6.4	Ref		
**History of TSI**						
yes	240	29	12.1	1.9	1.2-3.0	0.363
no	977	65	6.7	Ref		
**Number of partner**						
unique partner	941	51	5.4	Ref		
multiple partner	169	27	16	0.3	0.1-0.4	<0.001
**Multiple sexual intercourse of partner**						
yes	79	14	17.7	0.3	0.1-0.5	<0.001

not sure	1100	6	28.6	0.1	0.06-0.4
no	21	70	6.4	Ref	

Ref: reference category
